# Acute effects of wheel running on adult hippocampal precursor cells in mice are not caused by changes in cell cycle length or S phase length

**DOI:** 10.3389/fnins.2014.00314

**Published:** 2014-10-06

**Authors:** Tim J. Fischer, Tara L. Walker, Rupert W. Overall, Moritz D. Brandt, Gerd Kempermann

**Affiliations:** ^1^Genomics of Regeneration, CRTD – Center for Regenerative Therapies Dresden, Technische Universität DresdenDresden, Germany; ^2^Division of Neurodegenerative Diseases, Department of Neurology, Technische Universität DresdenDresden, Germany; ^3^Genomics of Regeneration, German Center for Neurodegenerative Diseases (DZNE) DresdenDresden, Germany

**Keywords:** adult neurogenesis, dentate gyrus, physical exercise, running, cell cycle, S phase

## Abstract

Exercise stimulates cellular brain plasticity by extending the pool of proliferating neural precursor cells in the adult hippocampus. This effect has been investigated extensively, but the most immediate cellular effect induced by exercise that results in this acute increase in the number of cycling cells remained unclear. In the developing brain as well as adult pathological models, cell cycle alterations have a major influence on the balance between proliferative and neurogenic divisions. In this study we investigated whether this might also apply to the acute physiological pro-neurogenic stimulus of physical exercise in adulthood. Do changes in cell cycle precede the measurable increase in proliferation? After 5 days of voluntary wheel running, however, we measured only a very small, statistically not significant acceleration in cell cycle, which could not quantitatively explain the observed increase in proliferating cells after exercise. Thus, at this acute stage, changes at the level of cell cycle control is not the primary causal mechanism for the expansion of the precursor cell population, although with time after the stimulus changes in cell cycle of the entire population of labeled cells might be the result of the expanded pool of cells that have progressed to the advanced neurogenic stages with shorter cell cycle length.

## Introduction

The fact that exercise has positive effects way beyond strengthening the muscles used is well known. In the brain it stimulates adult hippocampal neurogenesis—a complex process resulting in the generation of new functional neurons. More specifically physical exercise, which is experimentally achieved by free access to a running wheel, leads to a transient increase in proliferating neural precursor cells in the subgranular zone of the dentate gyrus (van Praag et al., 1999). While many studies have investigated the effects of genetics (Overall et al., 2013), kinetics (Kronenberg et al., 2006) or the functional relevance of this phenomenon (van Praag et al., 2005; Van der Borght et al., 2007), few have examined the immediate cellular effect leading to the pro-proliferative effect of running. There are several candidate mechanisms such as enhanced cell cycle entry via recruitment of previously quiescent stem cells, reduced cell cycle exit or modifications of cell cycle length itself. In the developing brain, cell cycle kinetics were identified as a major influence on the fragile balance between expansion and differentiation of neural progenitor cells (Lange et al., 2009). According to the cell cycle length hypothesis proposed by Calegari et al. (Calegari and Huttner, 2003), the length of the cell cycle or a certain phase defines the length of time a fate determinant acts—whatever its effect may be. In agreement with this idea, an artificially shortened G1 phase has been demonstrated to inhibit terminal neurogenic divisions but increase the proliferation of neural progenitor cells (Lange et al., 2009). This principle seems to apply to the adult mouse brain as well (Artegiani et al., 2011). Nevertheless, it has remained unclear whether changes at the level of cell cycle are equally causal for the physiologic response of the precursor cell pool in the adult hippocampus to extrinsic stimuli such as physical exercise (van Praag et al., 1999; Kronenberg et al., 2003). Studies of pathological models also suggest an important role of cell cycle modifications in adulthood: a combination of shortening of G1 and decreased cell cycle exit has been identified to cause the observed expansion of the proliferating cell pool after stroke (Zhang et al., 2008). Farioli-Vecchioli et al. have examined the impact of physical exercise on cell cycle kinetics under physiological conditions; they propose S phase acceleration as a key mechanism for the pro-proliferative effect after 12 days of free access to a running wheel (Farioli-Vecchioli et al., 2014). We know, however, that at this time point the pro-proliferative effect of exercise on the precursor cells peaks, returning to baseline levels in the following 3 weeks (Kronenberg et al., 2006). This left open the question, if changes in cell cycle would be cause or consequence of the exercise stimulus. Here, we thus addressed an earlier, more acute time point using a double labeling protocol (Brandt et al., 2012) to explore potential differences in total cell cycle and S phase length between standard housed mice and mice with free access to a running wheel.

## Materials and methods

### Animals and housing conditions

Female mice of the strain C57BL/6JRj were obtained from Janvier (France) and housed at the animal facility of Medizinisch-Theoretisches Zentrum at TU Dresden. During the experiment, animals were single-housed in standard cages with free access to water and food and a 12 h dark/light cycle. Mice assigned to the “Running” condition were provided with *ad libitum* access to a running wheel (150 mm diameter; TSE Systems, Germany) in their cage. All experiments were executed in accordance with the European and National regulations (Tierschutzgesetz) and approved by the responsible authority (Regierungspräsidium Dresden; Permit Number: 24-9168.11-1/2009-42).

### Experimental design

**Figure 1:**
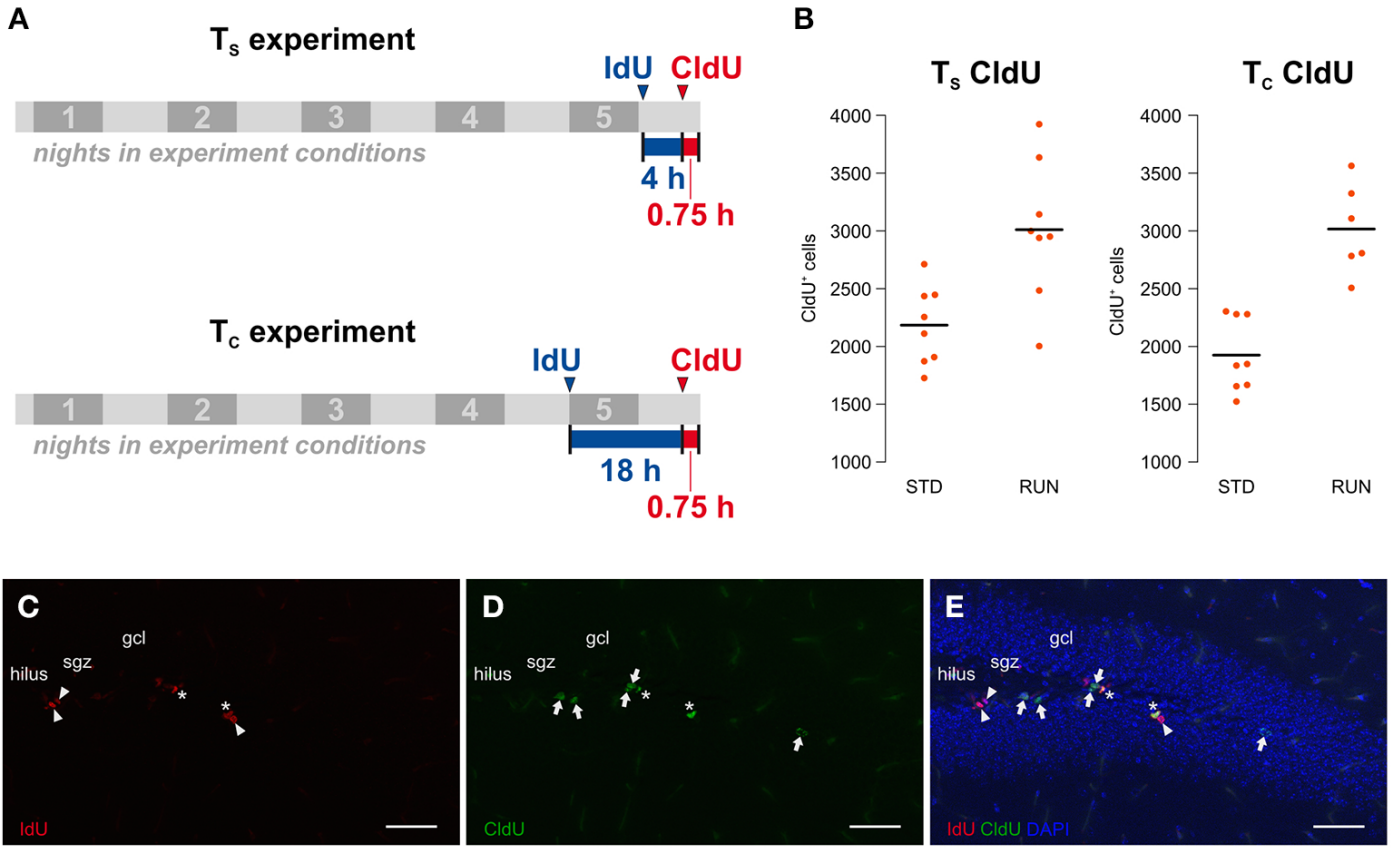
**(A)** Experimental design. The study consists of two experiments, including a runner group (RUN) and a standard housed group as control (STD) each, only differing in the time interval between the labeling. The T_S_ experiment aims to calculate the S phase length while the T_C_ experiment is designed for the calculation of the total cell cycle length on the basis of the calculated mean S phase length of the first experiment. 8 animals were assigned to each of the experimental groups. Mice were kept in experimental conditions for 5 nights and received an intraperitoneal CldU injection 45 min prior to their death. While animals in the T_S_ experiment were injected with IdU 4 h before the CldU injection, mice in the T_C_ experiment received this injection 18 h before the CldU injection. **(B)** Physical activity increases proliferation in both experiments. The total counts of CldU-labeled cells, indicating the size of the proliferating population, were measured in the two different environments “standard” (STD) and “running” (RUN). Animals of the RUN groups showed a significantly increased number of CldU^+^ cells compared to their controls in the T_S_ and the T_C_ experiment. **(C–E)** Injection intervals of 4 h and 18 h produced three differentially labeled populations: IdU^+^CldU^−^ cells (red, arrowhead; **C**) that were in S phase during the first injection only, IdU^−^CldU^+^ cells (green, arrow; **D**) that were in S phase during the second injection only and IdU^+^CldU^+^ cells (asterisk; **E**) that were in S phase during both injections. Scale bar 50 μm; sgz, subgranular zone; gcl, granule cell layer.

Our experiment was an adaptation of the protocol introduced by Brandt et al. (2012). Here we aimed to investigate potential changes in the cell cycle kinetics of proliferating hippocampal cells after 5 days of physical exercise. In principle, our study consisted of two experiments (Figure 1A) differing in the time interval between the labeling: one to calculate the length of the S phase (from here on referred to as the T_S_ experiment) and the second to calculate the total cell cycle length (from here on referred to as the T_C_ experiment). Both experiments involve both a runner group and a control group. A total of 32, 10 week-old mice were assigned to the 4 different experimental groups. The animals were single-housed in experimental conditions for 5 d before being killed. All animals received an intraperitoneal CldU injection (42.5 mg/kg) 45 min before their death, which is sufficient to label the majority of proliferative cells in S phase (Burns and Kuan, 2005). Additionally, mice from the T_S_ experimental groups received an IdU-Injection (57.5 mg/kg) 4:45 h before their death while mice from the T_C_ experimental groups received this injection 18:45 h before being killed. Thus, we used an inter-injection interval of 4 h for the T_S_ experiment and 18 h for the T_C_ experiment. Mice were killed by transcardial perfusion with 0.9% NaCl after receiving deep anesthesia by intraperitoneal ketamine (100 mg/kg) and xylazine (10 mg/kg) injection. An important general concern would be cross-reactivity between the IdU and CldU antibodies. However, the chosen protocol has been established for some time and cross-reactivity tests have been published previously (for example: Aten et al., 1992; Vega and Peterson, 2005; Brandt et al., 2012). In addition, brains labeled with both thymidine analogs and stained with both antibodies reveal cells that are detected only by one or the other antibody (see examples in Figure 1). This provides additional intrinsic confirmation that the antibodies do not indiscriminately detect both IdU and CldU.

### Tissue preparation

After dissecting the brain from the skull one randomly selected hemisphere was kept in 4% paraformaldehyde solution over night for post-fixation and then transferred to a 30% sucrose solution in 0.1 M phosphate buffer for 3–4 d. Using a Leica SM2010R microtome brains were cut into 40 μm thick sections in the coronal plane from rostral to caudal and transferred into cryoprotectant solution.

### Immunohistochemistry

Every sixth section of each brain was transferred into a multiwell plate containing phosphate buffered saline (PBS, pH 7.4). After several PBS and 0.9% NaCl washing steps the tissue was incubated with 2 M HCl at 37°C for 30 min for DNA denaturation, then washed again, blocked with PBS containing 10% donkey serum and 0.2% Triton X-100 and incubated with the primary antibodies overnight at 4°C. IdU was detected using mouse anti-BrdU antibodies (IgG; BD Biosciences), CldU by rat anti-BrdU antibodies (IgG; AbD Serotec) both at a concentration of 1:500. After several further PBS washing steps, the tissue was incubated with the secondary antibodies donkey anti-mouse CY3 (Jackson ImmunoResearch) and donkey anti-rat Alexa Fluor 488 (Jackson ImmunoResearch). Another washing step was followed by 4,6-diamidin-2-phenylindol (DAPI) incubation and further washing steps before the sections were mounted on slides in 0.1 M phosphate buffer and coverslipped using 2.5% PVA-DABCO.

### Quantification

In a complete series of 40 μm sections 240 μm apart, positively stained cells within the subgranular zone of the dentate gyrus were quantified in a blinded fashion using live imaging fluorescent microscopy on the Zeiss Apotome Axio Imager.Z1. All IdU and CldU single-labeled cells were counted. Additionally we phenotyped all IdU labeled cells in brain sections from the T_S_ experiment for a potential double staining with CldU. In specimens from the T_C_ experiment we phenotyped all CldU-positive cells for a potential double labeling with IdU.

### Statistics and calculations

Numerical analyses were performed using R (Version 3.0.2). Statistical differences were defined as significant at p = 0.05; 95% confidence intervals were used.

We calculated S phase length (T_S_) and total cell cycle length (T_C_) according to the method introduced by Brandt et al. (2012) but with inter-injection intervals of 4 h instead of 3 h for the T_S_ experiment and 18 h instead of 20 h or 16 h for the T_C_ experiment. Using cell counts of the T_S_ experiment we calculated the S phase length in the following way:

$$T_{S}\;=\;4h\;\times \;\frac{IdU^{+}}{IdU^{+}CldU^{-}}$$

Based on the mean S phase length of the respective group calculated in the T_S_ experiment and using cell counts of the T_C_ experiment, we then calculated the total cell cycle length as follows:

$$T_{C}\;=\;18\;h\;+\;\left(T_{S}\;\times \;\frac{IdU^{-}CldU^{+}}{CldU^{+}}\right)$$

T_S_ and $\frac{IdU^{-}CldU^{+}}{CldU^{+}}$ are both associated with statistical error terms. Therefore, statements regarding potential group differences of T_C_ require the use of an error propagation approach. For this purpose the distribution of the null hypothesis was calculated via 30 000 permuted calculations of potential group differences of T_C_. The *p*-value represents the probability of obtaining group differences of T_C_ at least as extreme as the ones we observed. For further information on the statistical method see Everitt and Hothorn (2009).

## Results

### Running increases proliferation in both the T_S_ and T_C_ experimental groups

Before examining potential changes in the cell cycle kinetics, one has to validate whether the experimental design revealed the expected pro-proliferative effect of running. Since CldU was injected 45 min prior to perfusion, cells in S phase at the end of our experiment were labeled as CldU-positive (CldU^+^) making them a relative surrogate for the absolute size of the proliferating population at this point in time. Total CldU^+^ cell counts were measured in the two different environments “standard” (STD) and “running” (RUN). As expected, we observed an increase in CldU^+^ cells after running in both the T_S_ [STD: 2184 ± 120, RUN: 3010 ± 213; mean ± s.e.m.; Welch Two Sample *t*-test; *t*_(11)_ = 3.38; *p* = 0.006] and T_C_ [STD: 1924 ± 112, RUN: 3016 ± 159; mean ± s.e.m.; Welch Two Sample *t*-test; *t*_(10)_ = 5.6; *p* = 0.00027] groups. Thus, we can conclude that our experimental setup is appropriate to investigate the exercise induced increase in proliferation. In a previous study with exactly the same set-up (but standard labeling with BrdU) we have also confirmed that this short-term running paradigm leads to an increase in the number of new neurons 4 weeks later (Overall et al., 2013).

### Physical activity does not affect S-phase or total cell cycle length

**Figure 2:**
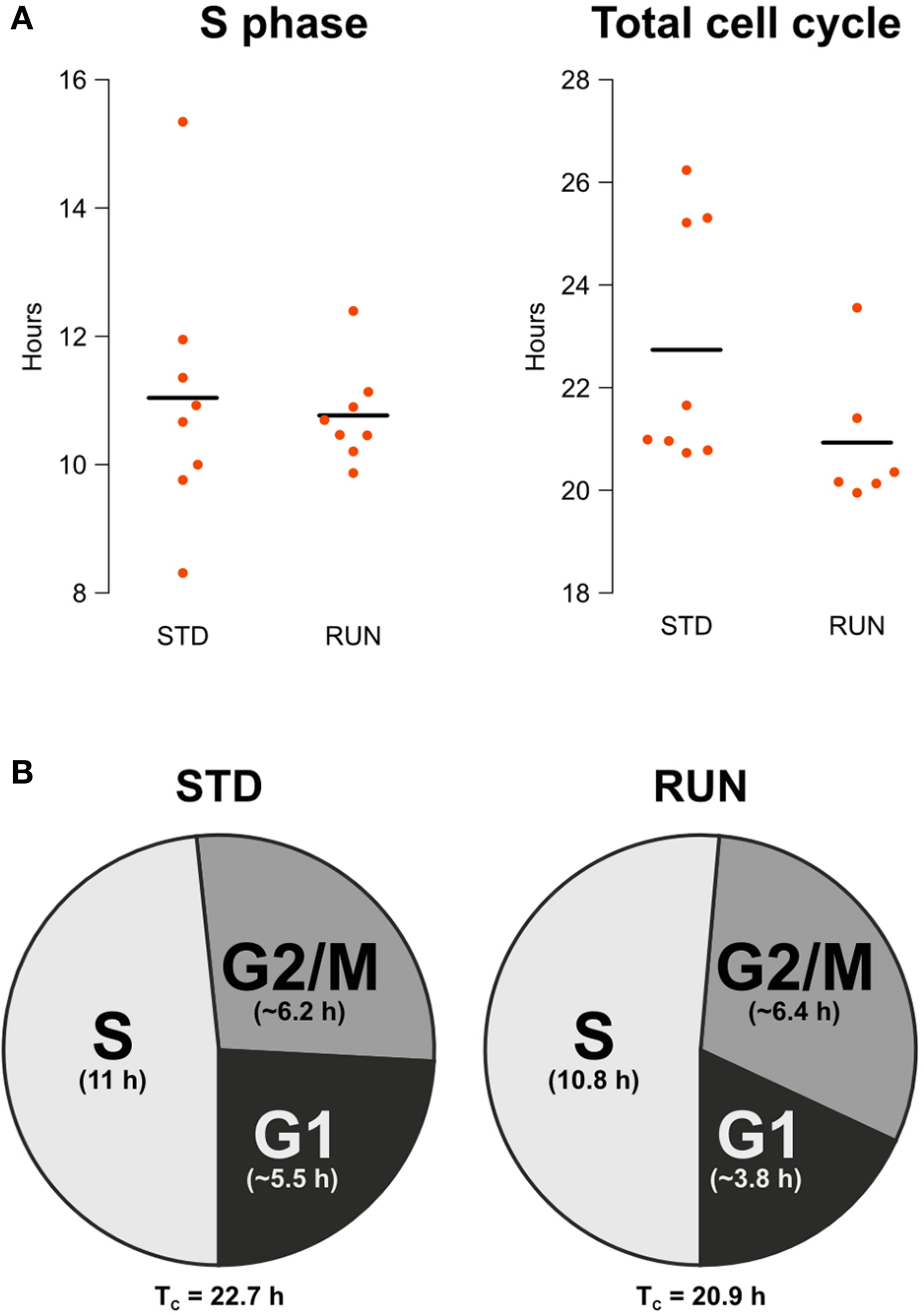
**(A)** S phase length and total cell cycle length are not affected by running. Beeswarm plot showing the parametrically calculated durations of T_S_ and T_C_ of every individual. **(B)** Composition of the cell cycle for animals housed in standard and running environments. Times for the total cell cycle and for the component stages are shown. G2/M and G1 phase lengths are given as the mean of the estimated range.

The main objective of our study was to examine potential changes in the length of the S phase or the entire cell cycle after physical exercise. We did not detect a change in S phase length [STD: 11.04 ± 0.73 h, RUN: 10.77 ± 0.27 h; mean ± s.e.m.; Welch Two Sample *t*-test; *t*_(9)_ = 0.35; *p* = 0.73] nor in total cell cycle length (STD: 22.74 h, RUN: 20.93 h, mean; permutation analysis; *p* = 0.1366; Figure 2A). Note that due to 2 dropouts the T_C_ RUN group consists of 6 animals as a result, while all other groups consisted of 8. The apparent outlier in the T_C_ RUN group remained in the analysis, since there was no substantiated reason to discount this animal and removal did not affect the outcome (data not shown).

### Estimation of G2/M length and G1 length

Besides an accurate calculation of S phase and total cell cycle length, the double labeling protocol we used also allows an approximate estimation of G2/M and G1 length. Comparing IdU^+^ and CldU^+^ cell counts within every mouse of the T_S_ experimental groups, i.e., the ones with an inter-injection interval of 4 h, the IdU^+^/CldU^+^ quotient was not significantly larger than 1 in both groups [standard: 1.128 ± 0.07; mean ± s.e.m.; One Sample *t*-test; *t*_(7)_ = 1.87; *p* = 0.103; runner: 1.12 ± 0.05; mean ± s.e.m.; One Sample *t*-test; *t*_(7)_ = 2.21; *p* = 0.063]. This means that within these 4.75 h before perfusion, the IdU-labeled population did not cycle through G2/M phase. Thus, G2/M is longer than 4.75 h in both experimental groups. Comparing IdU^+^ and CldU^+^ cells within the animals of the T_C_ experimental groups, i.e., the ones with an inter-injection interval of 18 h, the IdU^+^/CldU^+^ quotient was significantly larger than 2 in the standard group [IdU^+^/CldU^+^ = 2.39 ± 0.14; mean ± s.e.m.; One Sample *t*-test; *t*_(7)_ = 2.755; *p* = 0.028] but not in the runner group [IdU^+^/CldU^+^ = 2.22 ± 0.11; mean ± s.e.m.; One Sample *t*-test; *t*_(5)_ = 1.94; *p* = 0.11]. We expected the observed rough doubling of IdU^+^ cells compared to CldU^+^ cell counts in the 18 h inter-injection interval groups, because all IdU^−^labeled cells will pass through G2/M phase within the 18.75 h before perfusion.

This means:

$$T_{S}\;+\;G2/M\;<\;18.75h\text{ or }G2/M\;<\;18.75h-T_{S}$$

Therefore, G2/M is longer than 4.75 h and shorter than 7.71 h (~ 6.2 h) in the standard groups or 7.98 h (~ 6.4 h) in the runner groups. After calculating total cell cycle and S phase length and estimating G2/M length it is possible to estimate G1 as follows:

$$G1=T_{C}-T_{S}-G2/M$$

Thus, G1 length is between 3.99 and 6.95 (~ 5.5) h in the standard and 2.18–5.41 (~ 3.8) h in the runner groups (Figure 2B). Note that the estimates presented in the pie charts reflect only the means and do not take into account variance.

## Discussion

We designed this study to investigate potential acute changes in the kinetics of the cell cycle after physical exercise. The method employed allows precise determination of the length of the S phase and the total cell cycle. Additionally, one can also calculate estimations of the G2/M and G1 phase durations.

The key finding of our study is that there is no alteration in either the cell cycle length or the S phase length after 5 d of running. This is despite an average 47% increase in the proliferating cell population in the runners in comparison to the standard-housed mice.

Cell cycle kinetics have been studied extensively in the developing brain (Takahashi et al., 1995; Calegari and Huttner, 2003; Dehay and Kennedy, 2007; Lange et al., 2009). Here, cell cycle duration, more specifically G1 phase length, has been shown to influence the balance between proliferative and neurogenic divisions (Lange et al., 2009). A shorter G1 phase correlates with an increase in proliferation, while a prolonged G1 phase leads to a shift toward neurogenic divisions, i.e., more cells exiting the cell cycle after cleavage. To date, few studies have examined cell cycle alterations in the adult brain. A particularly well characterized model in this respect is the stroke model investigated by Zhang et al., revealing G1 shortening combined with a decrease in cell cycle exit as key mechanisms for the pro-proliferative effect of this pathological stimulus (Zhang et al., 2008). Note that these observations were made in the subventricular zone of rats and therefore might not apply equally to the subgranular zone of mice. We designed our study in order to examine potential cell cycle alterations after the physiological pro-proliferative stimulus of exercise. This issue has been addressed only once previously by Farioli-Vecchioli et al. (2014). Despite a very similar methodological approach, that study produced contradicting data—determining a reduction of S phase length by about 2.9 h and a subsequent shortening of the entire cell cycle as the key mechanism for increased proliferation. They detected a 23% increase in proliferating cell numbers after 12 days, a smaller effect than seen after 5 days of running in our study. This suggests that a more complex mechanism than simply an acceleration or shortening of the cell cycle is at work.

On a side note: our studies were done in single-housed mice, which despite these housing conditions showed a robust increase in cell proliferation after running. This is consistent with an independent previous study from our group (Overall et al., 2013), but in contrast to reports that social isolation in rats abolishes or at least delays the exercise-induced increase in precursor cell proliferation (Stranahan et al., 2006; Leasure and Decker, 2009).

Even though we did not detect a statistically significant acceleration of the cell cycle, our data reveal a trend toward a shorter cell cycle in the runner groups with a mean difference of 1.81 h. To put such potential reduction in cell cycle length into context, we can consider a model of division kinetics maximally affected by cell cycle acceleration. If we assume that:

1. the proliferation-promoting effect of physical exercise sets in immediately and acts homogenously over the 120 h mice spent in the experimental conditions, and
2. physical exercise does not induce any change at the level of cell cycle entry such as recruitment of previously quiescent stem cells, and
3. cell cycle exit is not altered, i.e., the same relative number of cells leave the proliferating population and proliferating cells and their progenies stay in the cell cycle for a certain time rather than for a certain number of divisions, in which case an accelerated cell cycle would decrease the proliferative population, and
4. all proliferating cells divide strictly symmetrically and generate two proliferating daughters during the 120 h experiment so that the proliferating population is maintained by a division model in which 2*^n^* cells originate from 1 precursor cell after *n* cell cycles, i.e., 2 cells after one cycle, 4 cells after two cycles, and so on, then

given the observed 1.81 h shortening of cell cycle in the runner group, cells in the standard housed mice would have to complete 5.29 cycles in the 120 h, while cells in the running mice would complete 5.74 cycles. In this extreme model, one cycling precursor cell would ultimately generate an average of 39.1 cells in standard housed mice and 53.4 cells in running mice. Thus, such a cell cycle acceleration after physical exercise would lead to a maximum 36.7% increase in proliferation given the extreme assumptions of this scenario. In reality, however, we observed a highly significant (*p* = 0.00027) 56.7% increase of CldU-labeled cells in the T_C_ running experimental groups. Thus, the theoretical expansion of the proliferating population via cell cycle shortening is not consistent with the actual data, even assuming a model of cell division that would be maximally affected by this acceleration.

**Figure 3:**
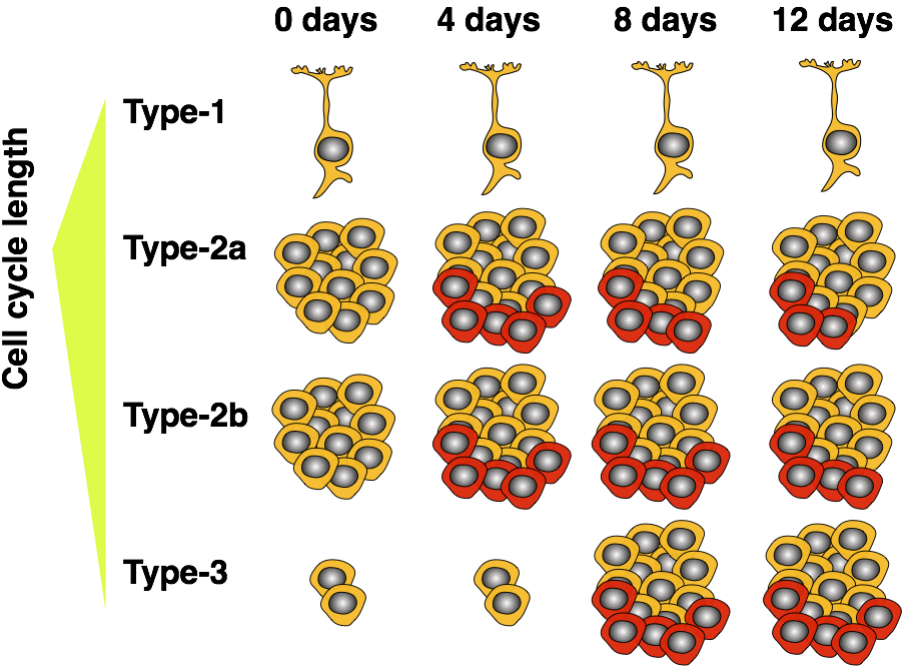
**Model showing how expansion of short cell cycle population influences average measured cell cycle length over time**. Highly proliferative type-2 cells are responsive to the running stimulus. As these mature, a proportionally larger population of late precursors (type-3) accumulates. The older, neuron-determined, cell stages have shorter cell cycles. As the expended population matures, the measured cell cycle length—averaged over all proliferating cells—decreases. Cells depicted in red indicate those additionally produced by wheel running.

Since our experiment investigated the cell cycle kinetics of the entire proliferating population, we cannot exclude the possibility of a differential response of the various cellular subpopulations—as claimed for GFAP^+^ and NeuroD1^+^ cells by Farioli-Vecchioli and colleagues in their study (Farioli-Vecchioli et al., 2014). However, we know that type-2 cells amount to roughly 90% of cycling cells in the dentate gyrus (Encinas et al., 2011) and are preferentially stimulated by running under acute conditions (Kronenberg et al., 2003; Steiner et al., 2008). We have also not detected exercise-induced effects on the proliferation of the radial type-1 cells (Steiner et al., 2004, 2008). Therefore, the observed and highly significant increase in proliferating cell numbers would strongly correlate with a shortened cell cycle of essentially the entire proliferating population if this was the main (and only) mechanism. Thus, for our data set we can exclude cell cycle acceleration as the sole mechanism leading to an exercise induced increase in neuronal proliferation after 5 d in running conditions. Yet one other explanation is also conceivable: As running strongly acts on type-2a cells, which have a longer cell cycle (Brandt et al., 2012), with progressing time, the enlarging fraction of proliferating type-2 cells will pass through the later precursor cell stages, which have a shorter total cell cycle length (Brandt et al., 2012). We have also previously shown that, after 7 days of running with proliferating cells labeled on day 5, the number of still proliferating cells decreased, while the proportion of doublecortin expressing cells was increased in the running animals—indicating a shift of currently dividing cells toward later precursor stages after 7 days of running (Brandt et al., 2010). Therefore, Farioli-Vecchioli et al. might not have observed an actual shortening of the mean cell cycle of the entire proliferating population leading to an increased size of this pool, but rather a relative shift toward later precursor cells. This model would suggest that a shortened cell cycle after running is not a cause, but rather an indirect consequence of the increased proliferation of early precursors (see schematic in Figure 3). Because our experiment focused on the acute, and thus more direct, effects of running, the smaller proportion of cells which had already progressed to later stages would explain the weak effect on average cell cycle length that we observed.

While it has been convincingly shown that manipulating the cell cycle is sufficient to affect neurogenesis in the embryo and the adult (Calegari and Huttner, 2003; Lange et al., 2009; Artegiani et al., 2011), it does not necessarily seem to be the case that physiological regulators only act through varying the length of the cell cycle. Our findings do not exclude that such effects might exist, but they cannot account for the entire exercise-induced increase in precursor cell proliferation. Our results presented here rather suggest that observed changes in cell cycle length after running may be a secondary consequence of the precursor pool expansion and not, as initially believed, a causal regulating factor.

## Author contributions

Tim J. Fischer, Moritz D. Brandt, Rupert W. Overall, Tara L. Walker, and Gerd Kempermann conceived and designed the experiments. Tim J. Fischer, Rupert W. Overall, and Tara L. Walker performed the experiments. Tim J. Fischer and Rupert W. Overall analyzed the data. Tim J. Fischer, Rupert W. Overall, Tara L. Walker, Moritz D. Brandt, and Gerd Kempermann wrote and edited the manuscript.

### Conflict of interest statement

The authors declare that the research was conducted in the absence of any commercial or financial relationships that could be construed as a potential conflict of interest.
